# DKK1 expression by synovial fibroblasts in very early rheumatoid arthritis associates with lymphocyte adhesion in an in vitro flow co-culture system

**DOI:** 10.1186/s13075-016-0915-3

**Published:** 2016-01-19

**Authors:** Maria Juarez, Helen M. McGettrick, Dagmar Scheel-Toellner, Lorraine Yeo, Julia Spengler, Banesa de Paz, Rowan Hardy, Mark Cooper, Karim Raza, Christopher D. Buckley, Andrew Filer

**Affiliations:** Rheumatology Research Group, Institute of Inflammation and Ageing (IIA), University of Birmingham, Queen Elizabeth Hospital, Birmingham, B15 2WB UK; Centre for Endocrinology, Diabetes and Metabolism, University of Birmingham, Birmingham, B15 2TT UK; ANZAC Research Institute, Concord Repatriation General Hospital, Concord, NSW 2139 Australia; Sandwell and West Birmingham Hospitals NHS Trust, Birmingham, B18 7QH UK; University Hospitals Birmingham NHS Foundation Trust, Birmingham, B15 2WB UK

**Keywords:** DKK1, Synovial fibroblasts, Early inflammatory arthritis, Lymphocyte adhesion

## Abstract

**Background:**

Synovial fibroblasts play a key role in joint destruction and regulation of the inflammatory infiltrate in established rheumatoid arthritis (RA). The mechanisms by which this occurs in the earliest stages of RA are largely unknown. We investigated the role of Dickkopf-related protein 1 (DKK1) produced by synovial fibroblasts of patients with very early rheumatoid arthritis (VeRA).

**Methods:**

Fibroblasts were isolated from the disease-modifying anti-rheumatic drug–naive Birmingham early arthritis cohort of patients with new onset of clinically apparent arthritis and inflammatory symptoms of ≤12 weeks’ duration, who at follow-up had either resolving arthritis or RA. Endothelial fibroblast co-cultures were formed using porous filters, and lymphocyte adhesion to co-cultures was assessed using phase-contrast microscopy. DKK1 gene expression and secretion were quantified by quantitative polymerase chain reaction and enzyme-linked immunosorbent assay, respectively.

**Results:**

Synovial fibroblasts from patients with VeRA expressed significantly higher levels of DKK1 messenger RNA than those from patients with resolving arthritis. A similar trend was observed for DKK1 protein secretion. In co-culture constructs, more DKK1 tended to be secreted in co-cultures incorporating fibroblasts from VeRA than in co-cultures from non-inflamed joints and resolving arthritis. DKK1 secretion during co-culture positively correlated with lymphocyte adhesion.

**Conclusions:**

Alterations in DKK1 could be involved in the pathogenesis and perpetuation of the inflammatory response in the earliest clinically apparent stages of RA.

## Background

Clinical studies have shown that early aggressive treatment of rheumatoid arthritis (RA) leads to better clinical outcomes without unacceptably high adverse event profiles [1]. The concept of an early window of opportunity is further supported by evidence of a distinct and transient cytokine profile in the synovial fluid of patients with RA of less than 3 months’ duration [2]. However, disease mechanisms during this very early stage of RA development remain largely undefined.

In established RA, multiple epigenetic changes drive the acquisition of a pathogenic phenotype in synovial fibroblasts that underpins their aberrant behaviour [3, 4]. This pathogenic phenotype is evidenced by the ability to invade human cartilage in a severe combined immunodeficiency model of arthritis [5, 6]. Additionally, rheumatoid synovial fibroblasts secrete proteases (e.g., matrix metalloproteinases and cathepsins) that degrade cartilage and bone tissue [7, 8]. Furthermore, they alter the dynamics of bone repair by releasing receptor activator of nuclear factor κB ligand (RANKL), causing osteoclast differentiation leading to bone erosion [9] whilst simultaneously producing Dickkopf-related protein 1 (DKK1) to inhibit osteoblast-driven repair of these erosions [10].

DKK1 is an inhibitor of the Wingless (Wnt) signalling pathway that has been proposed as a master regulator of joint remodelling [10]. Serum levels of DKK1 positively correlate with joint erosions and inflammation in RA [11]. Indeed, patients with a genetic variant of DKK1 which results in higher DKK1 serum levels have more progressive joint destruction [12], suggesting a fundamental role for DKK1 in the pathogenesis of RA. Treatment with antibodies against DKK1 has restored bone loss in murine models of arthritis [10], suggesting it has promise as a novel therapeutic target. In this study, to determine the role of DKK1 in the pathogenesis of early RA, we analysed, for the first time to our knowledge, the expression of DKK1 in synovial fibroblasts from patients with early arthritis that eventually resolved compared with patients whose arthritis developed into RA.

## Methods

### Patients

Synovial tissue samples were obtained by ultrasound-guided biopsy [13, 14] from patients recruited into the Birmingham early arthritis cohort (BEACON). BEACON is a cohort of disease-modifying anti-rheumatic drug (DMARD)-naive patients with clinically apparent synovitis in at least one joint and inflammatory joint symptoms (morning stiffness and/or inflammatory joint pain and/or swelling) of ≤12 weeks’ duration. Patients were assigned to one of two outcome categories at 18-month follow-up: resolving arthritis (*n* = 11) or RA (*n* = 14) (fulfilling 2010 American College of Rheumatology/European League Against Rheumatism [ACR/EULAR] criteria) [15]. Patients in the RA group are henceforth referred to as patients with very early rheumatoid arthritis (VeRA). Resolving arthritis was defined as no clinically apparent joint swelling with no DMARD or steroid use in the previous 3 months. In addition, healthy synovial tissue samples were collected from subjects undergoing exploratory arthroscopy for unexplained joint pain with no macro- or microscopic evidence of inflammation. Synovial fibroblasts were isolated as previously described [16] and used between passages 3 and 6 [17]. Demographic and clinical parameters, including age, sex, symptom duration, tender and swollen joint counts, erythrocyte sedimentation rate, C-reactive protein, rheumatoid factor and anti-cyclic citrullinated peptide status, were recorded.

### Quantification of DKK1

Fibroblast messenger RNA (mRNA) was extracted using the RNeasy Mini Kit (QIAGEN, Manchester, UK), and DKK1 expression was analysed by Applied Biosystems TaqMan low-density array (Life Technologies, Paisley, UK) using an Applied Biosystems 7900HT real-time polymerase chain reaction machine (Life Technologies). DKK1 levels were expressed relative to GADPH using the 2^−ΔΔCt^ method. DKK1 levels in serum and culture supernatants were quantified using the VersaMAP immunoassay or the DKK1 DuoSet enzyme-linked immunosorbent assay (R&D Systems, Abingdon, UK).

### Flow-based adhesion assay

Endothelial cells were isolated from umbilical cords as described elsewhere [18]. Endothelial fibroblast co-cultures were established on opposite sides of 0.4-μm-pore Transwell inserts (BD Biosciences, Oxford, UK) [17] for 24 h before treatment with 100 U/ml tumour necrosis factor-α (R&D Systems) and 10 ng/ml interferon-γ (PeproTech, London, UK) for a further 24 h. Filters were incorporated into a parallel plate chamber, and purified peripheral blood lymphocytes (2 × 10^6^ cells/ml) were perfused for 4 minutes as described elsewhere [17]. Digitised recordings were made of five random fields and analysed using Image-Pro Plus software (Media Cybernetics, Cambridge, UK). The number of adherent lymphocytes was averaged per field and expressed per square millimetre per 10^6^ cells perfused (Fig. 2b) [17].

### Ethical approval

All human samples were obtained with written informed consent and approval from the Human Biomaterial Resource Centre (Birmingham, UK), West Midlands and Black Country Research Ethics Committee or University of Birmingham Local Ethical Review Committee.

### Statistical analysis

Data analyses were performed using PASW 20.0 (SPSS, Chicago, IL, USA) and Prism 5 (GraphPad Software, La Jolla, CA, USA) software. Data were expressed as percentages, mean ± standard error of the mean and median (interquartile range) as appropriate. Two-group comparisons were performed using the χ^2^ test, unpaired *t* test and Mann-Whitney *U* test for categorical, parametric continuous and non-parametric continuous data, respectively. Three-group comparisons were performed with the Kruskal-Wallis test and Dunn’s post-test. Correlations were performed using Spearman’s test, where the *r* value relates to the non-parametric Spearman’s correlation coefficient. *p* Values less than 0.05 were considered statistically significant.

## Results

The demographic and clinical characteristics of patients in both outcome groups are shown in Table 1. Patients in the VeRA group were older, and a significantly higher number of them were female, compared with patients in the resolving group (9 vs. 3, respectively; *p* < 0.001). Patients with VeRA had more severe disease activity, as evidenced by higher tender joint counts (7.9 vs. 2.6, *p* = 0.02) and swollen joint counts (7.3 vs. 3.0, *p* = 0.04), higher disease activity scores (Disease Activity Score in 28 joints based on erythrocyte sedimentation rate 4.9 vs. 3.5, *p* = 0.004) and higher number of radiographic erosions at baseline (1 vs. 0, *p* < 0.001) than the patients in the resolving group. Seven of the fourteen patients in the VeRA group were undifferentiated at the time of biopsy (ACR/EULAR 2010 score <6). Fibroblasts from two male and two female individuals undergoing exploratory arthroscopy for unexplained joint pain with no macro- or microscopic evidence of inflammation were included as controls for co-culture recruitment assays (mean age 45.8 years, standard deviation 10.3).

**Table 1:** Demographic, clinical and laboratory characteristics of patients in each outcome group

	Resolving arthritis (*n* = 11)	VeRA (*n* = 14)	*p* Value
Age, yr	37.4 ± 10.3	52.9 ± 9.5	0.001
Female, *n* (%)	3 (27.3)	9 (64.3)	<0.0001
Disease duration, wk	4.4 ± 2.9	6.1 ± 3.3	0.210
CCP-positive, *n* (%)	0 (0)	7 (50)	<0.0001
RF-positive, *n* (%)	0 (0)	6 (42.9)	<0.0001
TJC28	2.6 ± 2.0	7.9 ± 5.8	0.008
SJC28	2.7 ± 2.0	7.3 ± 5.3	0.011
CRP	8.4 ± 8.1	23.0 ± 27.0	0.097
ESR	16.4 ± 15.5	31.1 ± 22.4	0.075
VAS	42.3 ± 32.1	46.9 ± 28.1	0.703
DAS28-ESR	3.5 ± 1.0	4.9 ± 1.2	0.004
Radiographic erosions, *n* (%)	0 (0)	1 (7.1)	<0.0001

*VeRA* very early rheumatoid arthritis, *CCP* cyclic citrullinated peptide, *RF* rheumatoid factor, *TJC28* 28-joint tender joint count, *SJC28* 28-joint swollen joint count, *CRP* C-reactive protein, *ESR* erythrocyte sedimentation rate, *DAS28-ESR* Disease Activity Score in 28 joints based on erythrocyte sedimentation rate, *VAS* visual analogue scale

Data are presented as mean ± standard deviation unless otherwise indicated

**Fig. 1:**
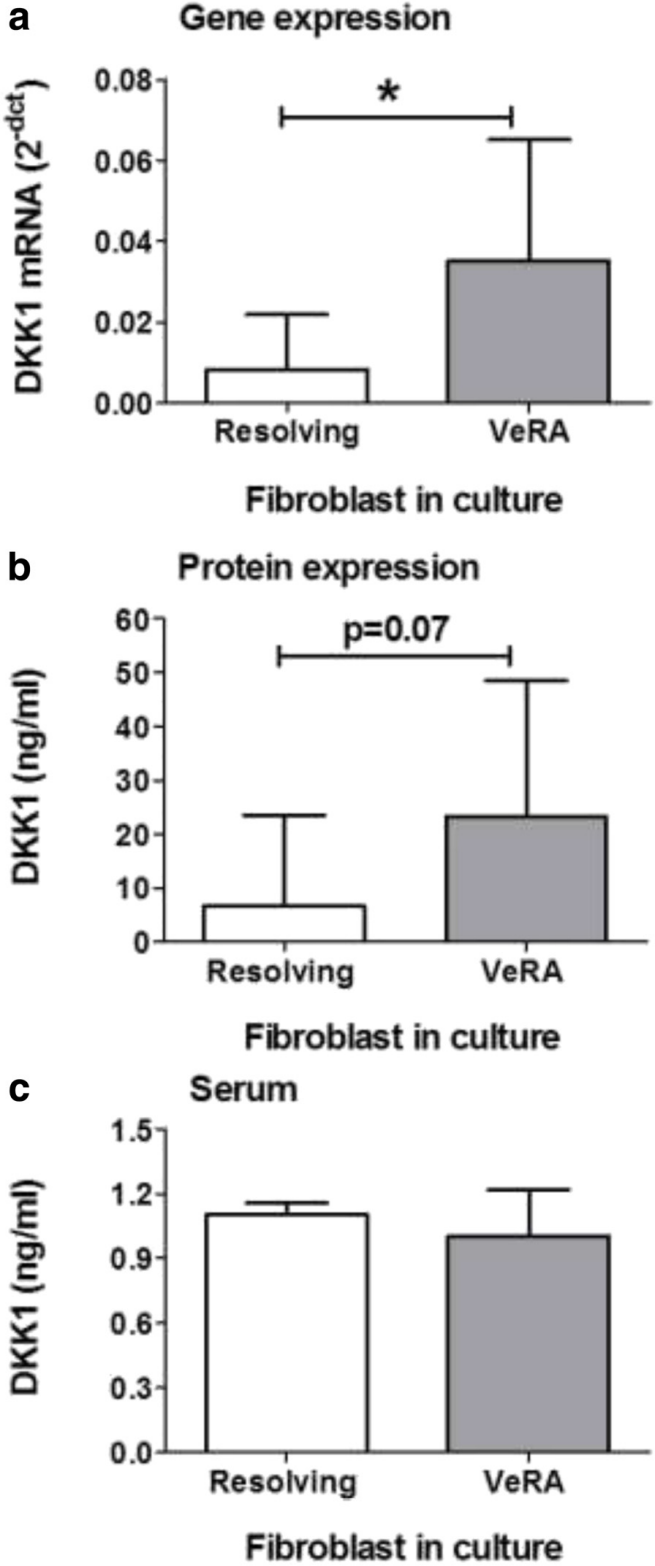
Dickkopf-related protein 1 (DKK1) expression in synovial fibroblasts. Gene expression (**a**) and secretion (**b**) of DKK1 were assessed in synovial fibroblasts isolated from patients with resolving arthritis or very early rheumatoid arthritis (VeRA). **c** Serum levels of DKK1 were assessed in patients with resolving arthritis or VeRA. Data are the median and interquartile range for 9 (resolving) and 12 (VeRA) independent experiments. *p < 0.05 by Mann-Whitney *U* test. *mRNA* messenger RNA

Synovial fibroblasts from patients with VeRA expressed significantly higher levels of DKK1 mRNA compared with those with resolving arthritis (Fig. 1a). A similar trend was observed for DKK1 secretion (Fig. 1b). Expression of DKK1 mRNA did not correlate with age, disease duration or any other clinical indices. There was no difference in DKK1 mRNA expression between patients with VeRA presenting with undifferentiated arthritis and those fulfilling criteria at presentation (data not shown). In contrast, serum levels of DKK1 were similar between the clinical outcome groups (Fig. 1c).

**Fig. 2:**
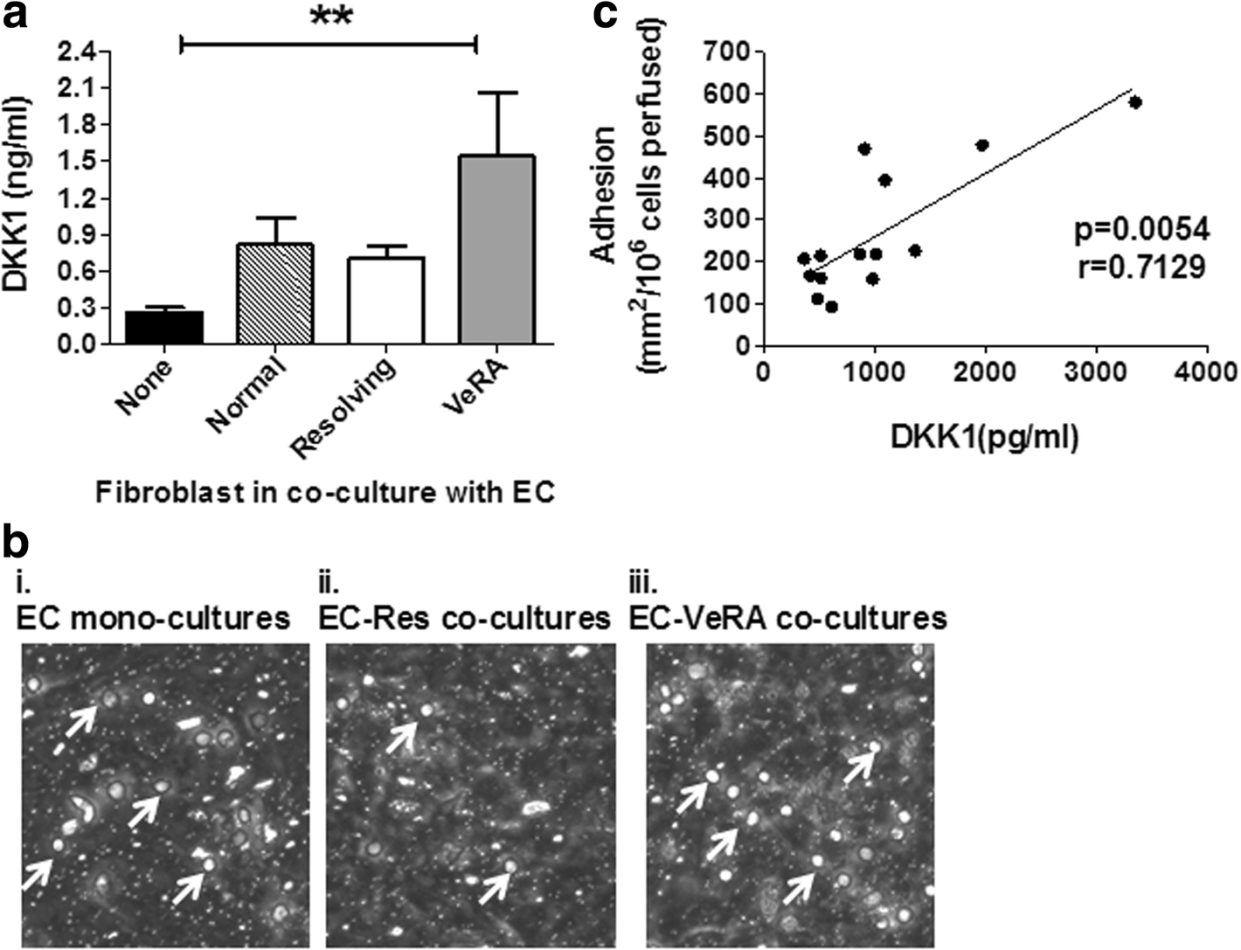
Dickkopf-related protein 1 (DKK1) levels in human umbilical vein endothelial cell (HUVEC) synovial fibroblast co-cultures. **a** DKK1 release from tumour necrosis factor (TNF)-α + interferon (IFN)-γ-treated endothelial cells cultured alone (none; *n* = 7) or with synovial fibroblasts from non-inflamed joints (*n* = 4), patients with resolving rheumatoid arthritis (RA; *n* = 5) or patients with very early rheumatoid arthritis (VeRA; *n* = 5). Significantly higher DKK1 levels were observed in the supernatant of VeRA-HUVEC co-cultures than HUVEC alone (*p* < 0.01). No statistical differences were found between the different co-culture constructs. Data are shown as mean and standard error of the mean. Kruskal-Wallis test with Dunn’s post-test analysis were performed. **p < 0.01. **b** Photomicrographs showing lymphocyte adhesion to (**i**) endothelial cells cultured alone, with fibroblasts from (**ii**) patients with resolving RA or (**iii**) patients with VeRA in response to TNF-α + IFN-γ treatment. The number of adherent lymphocytes was averaged per field and is expressed per square millimetre per 10^6^ cells perfused. *Arrows* indicate adherent lymphocytes. **c** DKK1 release positively correlated with lymphocyte adhesion to cytokine-treated co-cultures (*p* = 0.0054). Correlation assessed by Spearman’s test with a non-parametric correlation coefficient of *r* = 0.7129. Each independent experiment incorporated a different donor for all cell types

Next we assessed whether the differential expression of DKK1 in early disease had functional consequences. Endothelial fibroblast co-cultures were analysed for DKK1 secretion and for their ability to support lymphocyte adhesion from flow (Fig. 2b). In this model, more DKK1 was secreted in co-cultures incorporating fibroblasts from patients with VeRA compared with those from non-inflamed joints (normal) or patients with resolving disease, although this was not statistically significant (Fig. 2a). VeRA fibroblast co-cultures released significantly higher amounts of DKK1 than endothelial cells cultured alone (Fig. 2a). DKK1 secretion during co-culture showed a positive correlation with the level of lymphocyte adhesion supported by co-culture (Fig. 2c).

## Discussion

In this study, we examined, for the first time to our knowledge, the expression of DKK1 in synovial fibroblasts isolated from DMARD-naive patients with inflammatory arthritis of less than 3 months’ duration. Our data suggest that, even at this very early stage, fibroblasts from patients with VeRA may have gained the capacity to impair bone repair and induce bone erosion through increased expression of DKK1. Notably, this pathogenic phenotype was not observed in fibroblasts from patients with resolving arthritis where non-RA joint inflammation was also present. We also observed an association between DKK1 expression and the ability of fibroblasts from patients with VeRA to perpetuate the inflammatory response through lymphocyte adhesion. Whilst no causal relationship can be inferred, raised DKK-1 levels may help to provide an explanation for the long-observed association between inflammation and bone destruction. Collectively, these data indicate that DKK1 expression may be involved in early RA pathogenesis, through both the perpetuation of the inflammatory response and enhanced joint destruction.

DKK1 directly impairs osteoblast differentiation and indirectly enhances bone destruction by increasing RANKL-induced osteoclastogenesis [10, 19]. In established RA, expression of DKK1 within the synovium localises to synovial fibroblasts ex vivo [10] and is tightly regulated by glucocorticoid metabolism in vitro [20], supporting a role for Wnt signalling inhibition in RA bone destruction. In the work we present, differential expression of DKK1 in resolving and early persistent disease suggests that increased DKK1 production could be a key event in progression to RA and occurs early in the disease process. Wnt signalling inhibition by DKK1 may therefore be an as yet undefined pathway through which synovial fibroblasts influence bone destruction in early RA. Further work in this area should be directed towards confirmation of findings in in vivo models of arthritis.

## Conclusions

Synovial fibroblasts of patients with early inflammatory arthritis that persists as RA express higher DKK1 levels than those from patients with inflamed joints whose arthritis resolves. This phenomenon is amplified by co-culture with endothelial cells and associates with increased lymphocyte adhesion to co-cultures. We propose that DKK1 expression may be involved in early RA pathogenesis, through both perpetuation of the inflammatory response and enhanced joint destruction. Targeting of DKK1 may be a valid therapeutic approach early in the disease course.

## Abbreviations

ACR/EULAR: American College of Rheumatology/European League Against Rheumatism
BEACON: Birmingham early arthritis cohort
CCP: cyclic citrullinated peptide
CRP: C-reactive protein
DAS28: Disease Activity Score in 28 joints
DKK1: Dickkopf-related protein 1
DMARD: disease-modifying anti-rheumatic drug
ESR: erythrocyte sedimentation rate
HUVEC: human umbilical vein endothelial cell
IFN: interferon
mRNA: messenger RNA
RA: rheumatoid arthritis
RANKL: receptor activator of nuclear factor κB ligand
RF: rheumatoid factor
SJC28: 28-joint swollen joint count
TJC28: 28-joint tender joint count
TNF: tumour necrosis factor
VAS: visual analogue scale
VeRA: very early rheumatoid arthritis
Wnt: Wingless
